# Lack of Evidence on the Susceptibility of Ticks and Wild Rodent Species to PCV3 Infection

**DOI:** 10.3390/pathogens9090682

**Published:** 2020-08-21

**Authors:** Laura Grassi, Valentina Tagliapietra, Annapaola Rizzoli, Marco Martini, Michele Drigo, Giovanni Franzo, Maria Luisa Menandro

**Affiliations:** 1 Department of Animal Medicine, Production and Health (MAPS), University of Padua, 35020 Legnaro, Padua, Italy; laura.grassi.2@phd.unipd.it (L.G.); marco.martini@unipd.it (M.M.); michele.drigo@unipd.it (M.D.); marialuisa.menandro@unipd.it (M.L.M.); 2 Fondazione Edmund Mach, Research and Innovation Center, 38010 San Michele all’Adige, TN, Italy; valentina.tagliapietra@fmach.it (V.T.); annapaola.rizzoli@fmach.it (A.R.)

**Keywords:** PCV3, wildlife, wild rodents, ticks, reservoirs of infection, epidemiology

## Abstract

Porcine circovirus 3 (PCV3) is an emerging virus, first detected in 2016 and widespread in the swine industry. Although not considered a primary pathogen, PCV3 is potentially linked to several clinical conditions that threaten swine farming. Wild boars are considered the main reservoir species for PCV3 infection in the wild, but recent detection in roe deer, chamois and associated ticks has complicated our understanding of its epidemiology. Much emphasis has been placed on ticks, as competent vectors, and wild rodents, which typically feed immature tick stages. The aim of this study was to clarify whether wild rodent species and associated ticks are susceptible to PCV3 infection and involved in its spread. Wild small mammals’ serum samples and hosted ticks were, therefore, collected from areas where no wild boars were present and tested by PCR, targeting the PCV3 *rep* gene. A total of 90 yellow-necked mice (*Apodemus flavicollis*), two wood mice (*A. sylvaticus*), 26 bank voles (*Myodes glareolus*) and 262 *Ixodes* spp. ticks were investigated. PCV3 DNA was not detected in serum or in tick samples. These findings support the hypothesis that the investigated species do not have an actual role as PCV3 reservoirs. Further studies would be necessary to state whether these species, or others that we did not test, are involved in PCV3 infection spread—in particular when susceptible species share the same habitat.

## 1. Introduction

Porcine circovirus 3 (PCV3) is a small, single-stranded DNA (ssDNA) virus. It belongs to the family *Circoviridae,* genus *Circovirus*, characterized by a circular genome of approximatively 2 kb and nonenveloped virion [1,2].

PCV3 infects mammals of the *Suidae* family, primarily swine (*Sus scrofa domesticus*), but also wild boar (*Sus scrofa*) [1]. Prior to PCV3′s first identification in 2016, only two porcine circoviruses (PCVs) were known: PCV1, which seems to be nonpathogenic, and PCV2, which is among the most relevant viruses in swine farming [1,3]. Because of the negative impact that PCV2 has on the swine industry, many studies have focused on the emergence of PCV3, which has been rapidly detected worldwide. Additionally, retrospective studies highlighted that PCV3 has been circulating in swine farms since the 1990s [2]; the most recent common ancestor estimation, based on molecular clock hypothesis, dated PCV3′s origin back to well before the 20th century [4]. Thus, despite its recent identification, this virus cannot be considered a new pathogen; PCV3 has an ancient origin and circulated in the swine populations for decades [1,5].

In swine farms, this virus can infect animals of different ages and productive stages, being found in foetuses, stillbirths and new-borns, as well as in gilts, fattening pigs and multiparous sows [1,6]. Similar to PCV2, PCV3 has been detected in different clinical conditions, including reproductive, respiratory, gastroenteric and neurological disorders but also syndromes such as PDNS (porcine dermatitis and nephropathy syndrome) and systemic diseases [3,6,7,8,9]. However, it should be noted that PCV3 has also been found in asymptomatic animals [1,10]. Therefore, the wide range of symptoms and syndromes is likely not only the result of the virus itself, which is not considered a primary pathogen, but also of the occurrence of other predisposing factors, including environmental and host-related ones, as well as coinfections with other viral pathogens such as PCV2, porcine reproductive and respiratory syndrome virus (PRRSV) and porcine parvovirus (PPV) [1,5].

Another relevant susceptible species is the wild boar, in which, to date, a remarkably high prevalence (23%–44.8%) has been reported in apparently healthy animals [11,12]. PCV3 infects suidae mainly by horizontal transmission through direct contact and, even if less frequent, vertical transmission is also possible. Thus, considering the low density of wild boars compared to swine farms, viral transmission seems particularly efficient in this wild species, which is considered a PCV3 reservoir [1]. In fact, a recent longitudinal study conducted on asymptomatic wild boars (captured and recaptured) demonstrated that PCV3 infection persists for months [13]. Another, nonconflicting, hypothesis that could explain the high prevalence in the wild boar population [2,14] is the potential role of other wild animals and/or arthropods in the transmission and maintenance of PCV3 in the wild [5,11].

Accordingly, PCV3, similar to other PCVs, shows plasticity in host tropism and has also been reported in nonporcine species such as dogs, ruminants and mice [15,16,17,18]. Moreover, previous studies on PCV2 and PCV3 demonstrated that arthropods and rodents could also be involved in the spread of these viruses [11,19,20]. Recent findings showed that roe deer, mouflon, fallow deer and chamois tested positive for PCV3 [11,21], but also two PCV3-positive *Ixodes ricinus* ticks were collected from PCV3-negative roe deer [11], which opens up interesting questions about the role of these arthropods as vectors in the PCV3 enzootic cycle in nature. Since both ticks were adult females, and one of them was nonengorged, it was speculated that infection could have been acquired in previous life stages, which largely depend on small mammals [11,22].

Despite the role of small mammals being reported as a hypothesis [11], a recent review placed much emphasis on the role of rodents in PCV3 epidemiology and life cycle [5]. Due to the practical implications of an unfounded inference on PCV3 biology, we considered it of primary relevance to investigate PCV3 presence and frequency in wild rodents and their associated ticks, collected in areas where no wild boars were present and where, consequently, PCV3 was not previously reported. This could help to clarify whether wild rodents, directly or hosting immature stages of ectoparasites, play a role in PCV3 transmission and maintenance.

## 2. Results

**Figure 1:**
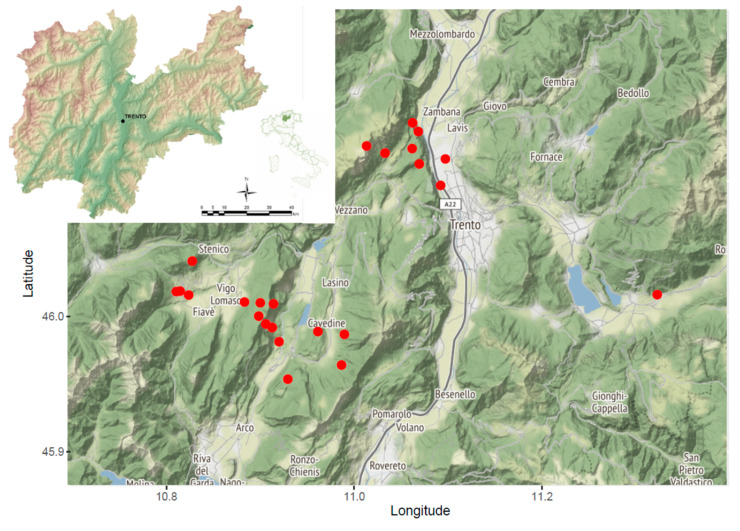
Map of the investigated area, where capture sites have been geolocalized. The position of the province of Trento in Italy is reported in the top-left insert.

Overall, a total of 257 serum samples from 118 individuals (90 *Apodemus flavicollis*, 2 *A. sylvaticus*, and 26 *Myodes glareolus)* captured between 2011 and 2012 in the province of Trento (Figure 1) and 395 associated tick samples were included in the study (see Table S1).

Sixty-three animals had one serum sample, while the remaining 55 had 2–9 sera samples available (Table 1). Based on the sample availability, and considering the previously demonstrated PCV3 assay specificity (i.e., 98%) and sensitivity (i.e., 94%) [23], the minimum expected prevalence at individual level allowing infection detection was 5%, accepting a 0.05 and 0.2 type I and II error, respectively.

According to the inclusion criteria (i.e., a maximum of three ticks for each developmental stage per animal), a total of 262 out of 395 ticks belonging to the genus *Ixodes* were analyzed—~10% at nymphal (*n* = 30) and ~90% at larval stages (*n* = 232). Viral DNA was not detected in any serum sample, whilst two tick larvae collected from yellow-necked mice tested positive, and one of them was engorged. However, no *Rep* or *Cap* sequence of adequate quality was obtained.

## 3. Discussion

After PCV3′s first detection in 2016, many studies have focused on its spread and pathogenic role in swine farms [1,3]. Most recently, other authors focused on wild boars, highlighting the remarkable prevalence in this wild species and supporting its probable role as a reservoir for PCV3 infection [11,12,13]. Then, more surprisingly, our previous study identified its genome in other ungulate species such as chamois and roe deer; additionally, PCV3 was detected in adult ticks collected from PCV3-negative roe deer [11]. Since one of the positive ticks was not engorged, a trans-stadial viral transmission was suggested. Considering that nymphal stages typically have their blood meal on small mammals, their susceptibility to PCV3 infection, and a potential epidemiological role, was hypothesized.

Furthermore, previous studies reported rodents as possible spreaders of PCV infection [19]. For instance, PCV2 was proven to infect mice in experimental conditions and also synanthropic rodents that lived close to swine farms, where they could have been infected due to the direct contact with positive swine [24,25,26]. To date, only one study has reported a considerable PCV3 prevalence in laboratory rodents, which neither had contact with swine populations nor were experimentally infected [16].

All this evidence is based on laboratory or synanthropic animals, likely exposed to a much higher infectious pressure, thus preventing any extension of these results to related wild species [16,19,24]. However, other authors have put emphasis on this potential part of the viral biological cycle and ecology based on our previous speculations [5].

Knowing the susceptibility of rodent species and ticks to PCVs and the actual lack of data in their wild counterparts, our study aimed to clarify their potential role as PCV3 reservoirs. We obtained several samples from areas where no wild boar populations were present, so we could state whether wild small mammals could play an active role regardless of contact with wild boar, thus allowing us to exclude incidental infections from the most likely reservoir.

All the analyzed serum samples were negative, in contrast with what was assumed by other authors in terms of the involvement of wild rodents in PCV3 spread [5]. At first, false negative results due to DNA degradation or inhibition were ruled out through the implementation of endogenous internal controls. The overall negative results suggest that the investigated species, yellow-necked mice (*Apodemus flavicollis*), wood mice (*Apodemus sylvaticus*) and bank voles (*Myodes glareolus*), do not act as PCV3 reservoirs in nature. The sample size represents the main study limitation; therefore, the PCV3 susceptibility of these species and a low level of circulation, below the set threshold of 5% prevalence, cannot be excluded. However, a PCV3 prevalence (if any) much lower than that described in wild boar and even other wild ungulates can be confidently stated [11,13].

Two out of 263 *Ixodes* ticks tested positive. However, different from our previous study, no sequence could be obtained and, in light of the specificity of the diagnostic test (i.e., 98%) and the low expected prevalence, the corresponding positive predictive value was low (approximately 30%). Therefore, according to the precautionary principle, we considered both of them false positives.

Based on these results, we hypothesize that the investigated species, yellow-necked mice (*Apodemus flavicollis*), wood mice (*Apodemus sylvaticus*) and bank voles (*Myodes glareolus*) do not play a pivotal role in PCV3 maintenance in nature and are not susceptible to PCV3 infection; moreover, ticks seemed not to be involved in PCV3 spread, at least when associated with the investigated wild rodent species. However, Franzo et al.’s detection of PCV3-positive ticks prevents us from drawing clear conclusions about the PCV3 infectious cycle [11].

We cannot exclude the possibility that other wild species could be involved in PCV3 epidemiology. Immature tick stages do not depend on small rodents alone, and other, nontested, hosts including birds, reptiles or other wild small mammal species typically feed larvae and nymphs [22,27,28]. Therefore, PCV3 susceptibility, if present, could likely affect only a subset of them, and further studies should be performed to increase the number of considered species and fill this knowledge gap.

In conclusion, PCV3 was not detected in the investigated wild small mammal sera or in ticks. These findings support the idea that the investigated species do not have an actual, direct or indirect, role in the PCV3 epidemiological cycle. Although largely contradicting the assumed role of small mammals and their associated ticks as PCV3 reservoirs, we do not exclude the possibility that the considered species, or other ones, could play a potential epidemiological role, at least as secondary hosts, when the presence of an actual reservoir guarantees an adequate infectious pressure. For this reason, further studies are currently ongoing to investigate the PCV3 presence in wild small mammals and ticks when sharing a habitat with PCV3-infected wild boar populations.

## 4. Materials and Methods

### 4.1. Sample Collection

Small mammal serum samples and related feeding ticks were provided by the Edmund Mach Foundation (Trento, Italy, http://www.fmach.it) and were a subset of a larger dataset obtained from several projects conducted in the Autonomous Province of Trento (Italy) on rodent and arthropod-borne pathogens. As a reference for the sampling design and collection procedures; see Rosà et al., 2019 [29]. All animal handling procedures and ethical issues were approved by the Wildlife Committee of the autonomous province of Trento (Prot. N. 595, issued on 4 May 2011). In more detail, ticks were selected if a blood sample from the respective small mammal had been collected. When available, multiple serum samples from the same animal were included in the study, even if no ticks were present, to follow them longitudinally and monitor viremia features, as performed in wild boars by Klaumann et al., 2019 [13].

Animals were captured in 2011–2012 in nine municipalities of the Autonomous Province of Trento, Italy (Figure 1, Table S2). Blood samples were centrifuged at 10,000 rpm for 12 min, and the serum was separated from the red blood cells and stored at −80 °C.

When present, tick specimens were collected from the animals, put in Eppendorf tubes with 70% ethanol and stored at room temperature. The number of ticks collected per animal varied from 1 to 13. A maximum number of three parasites at the same developmental stage per animal were analyzed. All ticks were processed individually.

Based on the sample availability, and considering the previously demonstrated PCV3 assay specificity (i.e., 98%) and sensitivity (i.e., 94%) [23], the sample size was determined to be able to detect at least one positive sample assuming a minimum expected prevalence of 5% at the individual level, accepting a 0.05 and 0.2 type I and II error, respectively. Based on these assumptions, the required sample size was 115 individuals. Overall, a total of 257 serum samples from 118 individuals (90 *Apodemus flavicollis*, 2 *A. sylvaticus* and 26 *Myodes glareolus)* captured between 2011 and 2012 in the province of Trento (from areas where no wild boars were present) and 395 associated tick samples were included in the study (Table S1).

### 4.2. PCV3 Detection

PCV3 infection diagnosis, targeting the *Rep* gene, was performed via the direct PCR assay previously described in [30]. Briefly, the Thermo Scientific Phire Animal Tissue Direct PCR Kit (Waltham, MA, USA) was used to perform PCR directly on biological samples, without previous DNA purification. To maximize the DNA availability, 2 µL samples of wild rodent serum or individual ticks were preprocessed according to the “dilution protocol”, as suggested by the kit manufacturer. The samples were incubated at room temperature for 5 min, followed by 98 °C for 4 min.

Samples were immediately used for PCR reactions using the primers and PCR protocol previously described in [30]. A swine serum sample previously demonstrated to be PCV3-positive was included in each run as a positive control. Negative controls were also included in each run. DNA release in ticks was confirmed by testing the preprocessed samples with a PCR protocol, targeting arthropods’ 16S gene, as described in Mangold et al., using the Thermo Scientific Phire Animal Tissue Direct PCR Kit [31]. Internal control sequencing was used for tick species characterization by comparing the obtained sequences to the reference ones available in GenBank through BLAST analysis. Reactions were performed in a final volume of 20 µL, containing 10 µL 2x Phire Animal Tissue PCR Buffer, 0.5 µL 16S forward and reverse primer, 0.4 µL Phire Hot Start II DNA Polymerase and 2 µL tick preprocessed sample. Sterile nanopure water was added until a volume of 20 µL was reached. The following thermal protocol was selected: 98 °C for 5 min, followed by 40 cycles of 98 °C for 5 s, 60 °C for 5 s and 72 °C for 10 s. A final extension phase of 72 °C for 1 min was also included.

Similarly, the successful extraction of DNA from serum samples was verified using the control primer mix provided by the Thermo Scientific Phire Animal Tissue Direct PCR Kit, according to the protocol suggested by the manufacturer. Internal control presence was tested in separate reactions, performed in parallel with the viral template search (Figure S1). All serum and tick samples were tested individually, both for the PCV3 and internal control detection.

PCR reactions were performed on an Applied Biosystems 2720 thermocycler (Applied Biosystems; Foster City, CA, USA). The amplification and specificity of bands were visualized using the SYBR Safe-stained 2% agarose gel (Figure S1). PCV3-positive samples were purified using the Thermo Fisher CleanSweep PCR Purification Reagent Kit, and DNA Sanger sequencing was attempted at Macrogen Europe (Amsterdam, The Netherlands). Additionally, *Cap* gene sequencing was also attempted following the protocol described in Franzo et al., 2019 [14].

## Figures and Tables

**Table 1:** Number of serum samples available per rodent species included in the study classified according to the species and number of times they have been captured and sampled. A = *Apodemus*; M = *Myodes*.

Number of Captures	*A. flavicollis*	*A. sylvaticus*	*M. glareolus*	Total
1	51	1	11	63
2	13		10	23
3	8	1	2	11
4	5		3	8
5	5			5
6	4			4
7	1			1
9	3			3
Total samples	90	2	26	118

## Supplementary Materials

The following are available online at https://www.mdpi.com/2076-0817/9/9/682/s1. Figure S1: Results of SYBR Safe-stained 2% agarose gel electrophoretic runs, representative of a set of endogenous internal control tests performed on rodent sera (a) and ticks (b). Table S1: List of animals analyzed with municipality of capture, total number of larvae and nymphs collected, total number of larvae and nymphs analyzed and number of sera samples collected (ID = sample number; A.FLAV = *Apodemus flavicollis*, A.SYLV = *A. sylvaticus*; M.GLA = *Myodes glareolus*). Table S2: Number of animals classified by species, per each sampling site (A = *Apodemus*; M = *Myodes*).
