# Supplementary material for: Lack of Evidence on the Susceptibility of Ticks and Wild Rodent Species to PCV3 Infection
Source: Pathogens. 2020 Aug 21;9(9):682. doi: 10.3390/pathogens9090682 (PMC7558181; doi:10.3390/pathogens9090682)
Supplement: Supplementary file 1 [file pathogens-09-00682-s001.zip › pathogens-900268-supplementary/Table S2.docx]

**Supplementary Table S2**. Number of animals classified by species, per each sampling site (A = Apodemus; M = Myodes).

|  | **A. flavicollis** | **A. sylvaticus** | **M. glareolus** | **Total** |
| --- | --- | --- | --- | --- |
| Cadine | 12 | 1 | 0 | 13 |
| Cavedine | 21 | 0 | 0 | 21 |
| Covelo | 9 | 1 | 0 | 10 |
| Lamar | 9 | 0 | 0 | 9 |
| Lundo | 12 | 0 | 11 | 23 |
| Panarotta | 9 | 0 | 0 | 9 |
| Pietramurata | 6 | 0 | 0 | 6 |
| San Giovanni | 1 | 0 | 1 | 2 |
| San Martino | 11 | 0 | 14 | 25 |
| Total | 90 | 2 | 26 | 118 |
