# Supplementary figures and images for: Lack of Evidence on the Susceptibility of Ticks and Wild Rodent Species to PCV3 Infection
Source: Pathogens. 2020 Aug 21;9(9):682. doi: 10.3390/pathogens9090682 (PMC7558181; doi:10.3390/pathogens9090682)

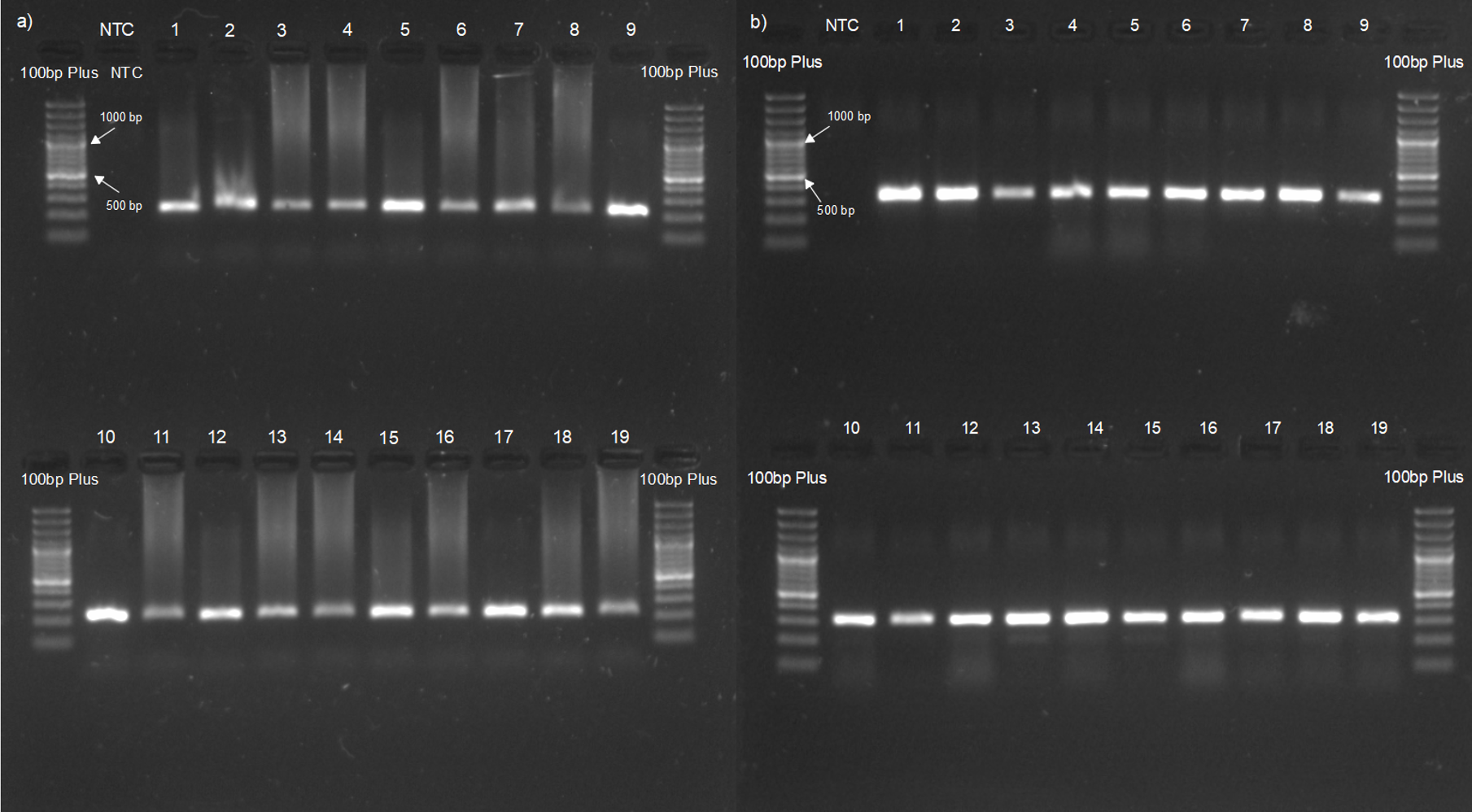

Supplement: Supplementary file 1 [file pathogens-09-00682-s001.zip › pathogens-900268-supplementary/Figure S1.png]
